# Percentage of Body Fat and Fat Mass Index as a Screening Tool for Metabolic Syndrome Prediction in Colombian University Students

**DOI:** 10.3390/nu9091009

**Published:** 2017-09-13

**Authors:** Robinson Ramírez-Vélez, Jorge Enrique Correa-Bautista, Alejandra Sanders-Tordecilla, Mónica Liliana Ojeda-Pardo, Elisa Andrea Cobo-Mejía, Rocío del Pilar Castellanos-Vega, Antonio García-Hermoso, Emilio González-Jiménez, Jacqueline Schmidt-RioValle, Katherine González-Ruíz

**Affiliations:** 1 Centro de Estudios para la Medición de la Actividad Física CEMA, Escuela de Medicina y Ciencias de la Salud, Universidad del Rosario, Bogotá DC 111221, Colombia; jorge.correa@urosario.edu.co (J.E.C.-B.); alesanders_0615@hotmail.com (A.S.-T.); 2 Grupo CORPS, Universidad de Boyacá, Facultad de Ciencias de la Salud, Boyacá 150003, Colombia; mlojeda@uniboyaca.edu.co (M.L.O.-P.); eacobo@uniboyaca.edu.co (E.A.C.-M.); dpcastellanos@uniboyaca.edu.co (R.d.P.C.-V.); 3 Laboratorio de Ciencias de la Actividad Física, el Deporte y la Salud, Facultad de Ciencias Médicas, Universidad de Santiago de Chile, USACH, Santiago 7500618, Chile; antonio.garcia.h@usach.cl; 4 Departamento de Enfermería, Facultad de Ciencias de la Salud, Avda. De la Ilustración, 60, University of Granada, 18016 Granada, Spain; emigoji@ugr.es (E.G.-J.); jschmidt@ugr.es (J.S.-R.); 5 Grupo CTS-436, Adscrito al Centro de Investigación Mente, Cerebro y Comportamiento (CIMCYC), University of Granada, 18071 Granada, Spain; 6 Grupo de Ejercicio Físico y Deportes, Vicerrectoría de Investigaciones, Universidad Manuela Beltrán, Bogotá DC 110231, Colombia; katherine.gonzalez@docentes.umb.edu.co

**Keywords:** obesity, adiposity, fat mass, metabolic syndrome

## Abstract

High body fat is related to metabolic syndrome (MetS) in all ethnic groups. Based on the International Diabetes Federation (IDF) definition of MetS, the aim of this study was to explore thresholds of body fat percentage (BF%) and fat mass index (FMI) for the prediction of MetS among Colombian University students. A cross-sectional study was conducted on 1687 volunteers (63.4% women, mean age = 20.6 years). Weight, waist circumference, serum lipids indices, blood pressure, and fasting plasma glucose were measured. Body composition was measured by bioelectrical impedance analysis (BIA) and FMI was calculated. MetS was defined as including more than or equal to three of the metabolic abnormalities according to the IDF definition. Receiver operating curve (ROC) analysis was used to determine optimal cut-off points for BF% and FMI in relation to the area under the curve (AUC), sensitivity, and specificity in both sexes. The overall prevalence of MetS was found to be 7.7%, higher in men than women (11.1% vs. 5.3%; *p* < 0.001). BF% and FMI were positively correlated to MetS components (*p* < 0.05). ROC analysis indicated that BF% and FMI can be used with moderate accuracy to identify MetS in university-aged students. BF% and FMI thresholds of 25.55% and 6.97 kg/m^2^ in men, and 38.95% and 11.86 kg/m^2^ in women, were found to be indicative of high MetS risk. Based on the IDF criteria, both indexes’ thresholds seem to be good tools to identify university students with unfavorable metabolic profiles.

## 1. Introduction

With increasing prevalence worldwide, obesity has become a significant public health issue [1]. Obesity has been linked to a number of cardiovascular disease (CVD) risk factors including metabolic syndrome (MetS) [2]. In Colombia, it is estimated that over 50% of adults are overweight or obese and the obesity epidemic has been associated with obesogenic factors, such as an intake of energy-dense diets, a sedentary lifestyle, and low levels of physical activity [3,4] .

In this context, there is growing evidence that suggests that the way in which fat is distributed contributes different effects on the cardiometabolic risk associated with obesity [3,4,5]. In addition, measures of central body obesity, such as waist circumference and waist–hip ratio, have been suggested as being more strongly related to MetS risk compared to body mass index (BMI) [4,5]. Aside from age or sex, excess body fat is the strongest determinant of an individual’s risk of developing MetS [6], and several epidemiological studies have found an association between fat distribution and metabolic risk factors, including high blood pressure, dysglycemia, dyslipidemia, and subsequent risk of MetS [4,5].

Although BMI is the most frequently used method to assess the level of obesity [7], BMI does not differentiate between body lean mass and body fat mass; that is, a person can have a high BMI but still have a low fat mass and vice versa [8,9]. In addition, the ongoing uncertainty as to which measure of body fat is most important at gauging an individual’s risk [6], particularly with respect to MetS, may also contribute to the lack of their use and monitoring in clinical practice.

In this sense, Computed Tomography (CT) or dual-energy X-ray absorptiometry (DXA), continue to be the gold standard for evaluating the distribution of body fat [10]. Nevertheless, the high cost and low availability have made it difficult to use in large population studies. Evidently, this factor limits the possible screening of MetS in high-risk populations, especially in developing countries such as Colombia [2,11]. Recently, more research has examined the potential role of body composition measurements in health monitoring [12,13]. Bioelectrical impedance analysis (BIA) is the method that is most frequently used to assess body composition and calculate BF% in clinical practice, given its accuracy, simplicity, low cost, and excellent correlation with DXA, CT, or magnetic resonance imaging (MRI) [14,15].

Alternative simple and inexpensive approaches for assessing body adiposity have been suggested. A recent study highlighted the usefulness of the fat mass index (FMI), measured as kg/m^2^, as an indicator of the function of adipose tissue as a surrogate marker of cardiovascular risk in young adults [16]. However, BF% and FFM are known to change with height, weight, sex, and age. Along this line, VanItallie et al. [16] proposed an FMI that considers an individual’s height. FMI, like BMI except that it uses a two-compartment model, merits reappraisal and appears to be of interest in the classification of overweight, obese, and underweight patients [17]. Previous research has evaluated the applicability of FMI in the prediction of MetS and has highlighted its close relation with the components of MetS [17]. However, only a few studies have focused on the differential impacts adiposity levels [18,19,20,21,22,23,24] in young adults when examining the link between fat and the risk of MetS.

Understanding the specific fat distribution associated with MetS risk is important to improve the interventions that are currently more focused on overall weight loss [5,6]. Our study proposes a gender-specific fat index based on BIA as a way of estimating the fat mass and body adiposity dysfunction associated with MetS. Apart from the study of Liu et al. [17], this is the first study in Colombian young adults (university students) to investigate the association between fat mass by BIA and MetS components. Based on the IDF definition of MetS [2], the aim of the study was to explore thresholds of BF% and FMI for the prediction of MetS among Colombian university students.

## 2. Methods

### 2.1. Study Design and Sample Population

During the 2014–2017 academic years, we reviewed a cross-sectional component of the FUPRECOL study that investigated the association between muscular strength and metabolic risk factors in Colombian collegiate students. The FUPRECOL study aimed to establish the general prevalence of cardiovascular risk factors, including anthropometric and metabolic markers, in the study population and to examine the relationships between physical fitness levels, body composition, and cardiometabolic risk factors. We recently published a complete description of the FUPRECOL study design, methods, and primary outcomes for our current cohort [2]. The sample consisted of adults (men: *n* = 707; women: *n* = 1128). We removed cases due to missing (*n* = 37, 2.0%) or erroneous data entry (*n* = 83, 4.5%) and those which had neither age nor a valid date of birth recorded (*n* = 28, 1.5%), limiting the analytical sample to 1687 volunteers, which was 63.4% women and a mean age of 20.6 years. The subjects, whose ages ranged from 18 to 35 years, were all of low to middle socioeconomic status (SISBEN levels: 1–4 on a scale of 1–6 as defined by “The System of Identifying Potential Beneficiaries of Social Programs”, called SISBEN). The system takes into account sociodemographic characteristics including family composition, employment status, family income, and educational level; living conditions, including construction type and materials; and access to public utilities, which involves sewers, electricity, potable water, and garbage collection. They were enrolled in public or private universities in the capital district of Bogota, Boyacá, and Cali, Colombia.

Exclusion criteria included the following: medical or clinical diagnosis of a major systemic disease including malignant conditions such as cancer, type 1 or 2 diabetes, high blood pressure, hypothyroidism or hyperthyroidism; a history of drug or alcohol abuse; regular use of multivitamins; chronic inflammatory conditions including rheumatoid arthritis, systemic lupus erythematosus, multiple sclerosis; infectious conditions; and a BMI ≥ 35 kg/m^2^. Volunteers received no compensation for their participation.

### 2.2. Data Collection

Subjects were screened for inclusion in the study via personal interviews. Interview questions collected consisted of health status, medical history, CVD risk factors, and lifestyle. After completing another general information questionnaire, participants were instructed to wear shorts and a T-shirt to the physical exam. They were also required to remove all worn jewelry and metal objects. Once the subjects were barefoot and in their underwear, their body weight (kg) was measured using an electric scale (Model Tanita^®^ BC-418^®^, Tokyo, Japan) with a range of 0–200 kg and with an accuracy of within 100 g. Height was measured with a portable stadiometer with a precision of 0.1 cm and a range of 0–2.5 m (Seca^®^ 274, Hamburg, Germany). BMI was calculated by using the formula proposed by Quetelet where BMI = body mass (kg)/height (m^2^). Body mass index status was evaluated according to the World Health Organization (WHO) criteria [24]. The waist circumference (WC) (cm) was measured as the narrowest point between the lower costal border and the iliac crest. When this point was not evident, it was measured at the midpoint between the last rib and the iliac crest, using a metal tape measure (Lufkin W606PM^®^, Parsippany, NJ, USA), in accordance with the International Society for the Advancement of Kinanthropometry guidelines [25]. The evaluation process was carried out by a team of professionals (four physical therapy professors) with extensive experience in anthropometric measurement. Two percent of the sample was measured twice in order to ensure quality of measures. The technical error of measurement (TEM) values was less than 2% for all anthropometric variables.

BF% and FMI were determined for BIA by a tetrapolar whole body impedance (Tanita Model BC-418^®^, Tokyo, Japan). A detailed description of the BIA technique can be found in a previous study [2]. For the calculation of intra–inter observer TEM, at least 50 subjects needed to be measured (30 men, 20 women, aged 22.3 ± 2.1 years). The corresponding intra-observer technical error (% reliability) of the measurements was 95%. FMI was then calculated by dividing each subject’s fat mass (kg) by the square of his or her height (m), as previously described [17]. In a sub-sample (48 adults, 54% women, range mean age = 20 to 40 years old), we verified the validity of BIA for predicting BF% in Colombian adults using DXA as a reference. Our analysis showed a strong agreement between the two methods, as reflected in the range of BF% (Lin’s concordance correlation coefficient = 0.943 (95% CI = 0.775 to 0.950, *p* = 0.041) and bias −0.6 (SD 2.2; 95% CI = −5.0 to 3.7). In line with our findings, a previously study has shown a high correlation between fat mass determined by BIA and that obtained by a CT scan and DXA [14,15,26]. Thus, these results show that BIA and DXA are comparable methods for measuring body composition with lower/higher body fat percentages.

### 2.3. Metabolic Syndrome Diagnosis

After the subjects had fasted for 10–12 h, blood samples were obtained from capillary sampling between 6:00 a.m. and 9:00 a.m. Participants were asked not to engage in prolonged exercise in the 24 h prior to testing. The biochemical profile included the following: (i) high-density lipoprotein cholesterol (HDL-C); (ii) triglycerides; (iii) low-density lipoprotein cholesterol (LDL-C); (iv) total cholesterol; (v) glucose fasting by enzymatic colorimetric methods (CardioChek PA^®^, Polymer Technology Systems, PTS, Indianapolis, IN, USA). The inter-assay reproducibility (coefficient of variation) was determined from 16 replicate analyses of 8 capillary blood pools over a period of 15 days. The percentages obtained were 2.6% for triglycerides, 2.0% for total cholesterol, 3.2% for HDL-C, 3.6% for LDL-C, and 1.5% for fasting glucose.

Blood pressure was taken on the left arm at the heart level with an automatic device Omron M6 Comfort (Omron^®^ Healthcare Europe B.V., Hoofddorp, The Netherlands) while the participants were sitting still. The blood pressure monitor cuff was placed two to three finger-widths above the bend of the arm and a two-minute pause was allowed between the first and second measurements with a standard cuff for an arm circumference of 22–32 cm.

MetS was defined in accordance with the updated harmonized criteria of the IDF [2]. Participants were considered to have MetS if they showed three or more of the following: (1) abdominal obesity for individuals (WC ≥ 80 cm in women and ≥ 90 cm in men) [27]; (2) hypertriglyceridemia (≥150 g/dL); (3) low HDL-C (<50 mg/dL in women and <40 mg/dL in men); (4) high blood pressure (systolic blood pressure ≥ 130 mmHg or diastolic blood pressure ≥ 85 mmHg); (5) high fasting glucose (≥100 mg/dL).

### 2.4. Lifestyle Co-Variables

A standardized questionnaire, FANTASTIC lifestyle, was used to collect comprehensive information about substance use via a personal interview with participants [28]. Alcohol consumption and smoking status were defined as subjects who had consumed any alcoholic beverage ≥1 times per week, and those who had smoked ≥10 cigarettes per week, for at least six months, as previously described by Ramírez-Vélez et al. [28]. Participants who exercised three or more times a week for >30 min were categorized as physically active (PA), and those who exercised less than three times a week were considered insufficiently physically active. The accuracy of information about lifestyle co-variables obtained from the FANTASTIC questionnaire has been validated by different cross-sectional studies and described in detail elsewhere [28,29].

### 2.5. Ethics Statement

Informed consent was obtained from each participant. The protocol was based on the Helsinki Declaration Accord (World Medical Association for Human Subjects). Moreover, ethical approval was obtained from the Universidad Manuela Beltrán (UMB N° 01-1802-2013).

### 2.6. Statistical Analysis

Participants’ characteristics obtained were given as mean values and standard deviation (SD). Histograms and Q–Q plots were used to verify the normality of the selected variables. Independent two-tailed *t*-tests for continuous variables, and chi-square (χ^2^) tests for categorical variables, were used to examine sex differences or MetS grouping. The relationships between BF%, FMI, and MetS components were tested by means of partial correlation coefficients. This analysis was adjusted by age, sex, tobacco, and alcohol consumption, and PA levels. To predict MetS with BF% and FMI, we used area under the curve (AUC), ranging between 0 and 1 (a worthless and a perfect test, respectively), which is a global indicator of diagnostic performance, and represents the ability of the test to correctly classify participants with high risk MetS by *p*-values < 0.01 and an AUC > 0.80. The positive likelihood ratio LR (+) and the negative likelihood ratio LR (−) were also determined. Cutoff points were chosen based on the highest Youden index, i.e., the point on the receiver operating characteristic curve (ROC) that is farthest from the line of equality [30]. Data analysis was performed using the Statistical Package for the Social Sciences for Windows SPSS, version 21.0 (SPSS Inc., Chicago, IL, USA). Statistical significance was set at *p* < 0.05.

## 3. Results

### 3.1. Descriptive Characteristics

Descriptive characteristics of the participants are presented in Table 1. The final sample had a mean age of 20.6 years (SD 3.0; range 19–24 years), with 63.4% of participants being women. Women were found to have significantly lower height, WC, blood pressure, triglycerides, and PA than men (*p* < 0.05). The prevalence of MetS was higher in men than in women at 11.2% vs. 5.3% (*p* < 0.001).

**Figure 1:**
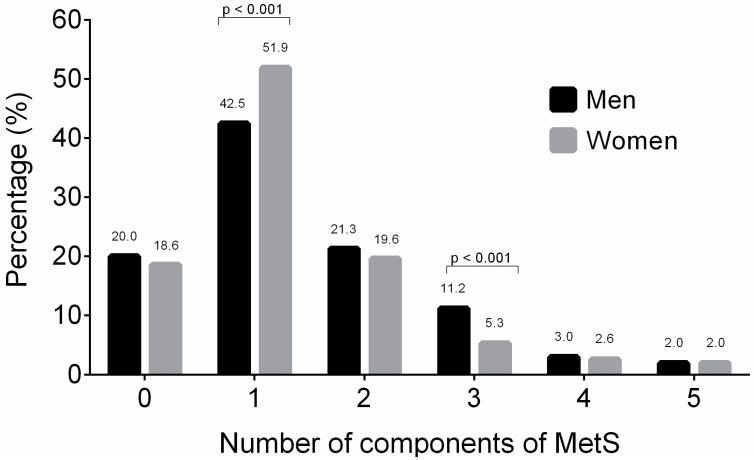
Distribution of the prevalence of metabolic syndrome components according to sex.

At least one MetS component was found in 818 participants (48.5%), two MetS components were present in 341 participants (20.2%), three MetS components were found in 126 participants (7.7%), and four or more components of MetS were present in 80 participants (4.7%) (Figure 1).

### 3.2. Clinical Characteristics and Distribution by MetS Status

Independent of sex, participants with MetS had significantly higher weight, body mass index, WC, BF%, FMI, and total cholesterol and triglyceride levels (*p* < 0.01) (Table 2). Furthermore, participants without MetS were more active (34.5% vs. 17.8% for men, and 21.1% vs. 6.8% for women, *p* < 0.01).

Table 3 shows the partial correlation between BF%, FMI, and MetS components. Overall, BF% and FMI were weakly positively correlated with all MetS parameters (all *p* < 0.05), except HDL, which was negatively correlated.

### 3.3. Optimal Cut-Off Value in the Screening of MetS

**Figure 2:**
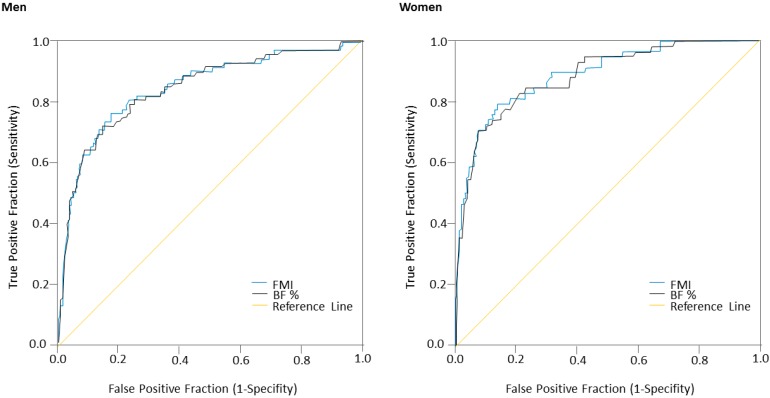
ROC curves for BF% and FMI for prediction of the prevalence of MetS according to IDF criteria in men and women.

The ROC analysis showed that BF% and FMI parameters could be used to detect MetS according to the IDF criteria among Colombian university students (Table 4; Figure 2). Since multiple levels of risk were desired, the selection of the resulting cut-off values was based on multiple combinations of sensitivity and specificity. The focus was on higher sensitivity for the BF% and FMI thresholds in an effort to identify the majority of cases of MetS according to the IDF criteria. In men, the cut-off point value of 25.5% for BF% provided a sensitivity of 96.1%, an LR (+) value of 2.3, a specificity of 57.5%, and an LR (−) value of 0.06. For FMI, the cut-off value of 6.9 kg/m^2^ provided a sensitivity of 95.8%, an LR (+) value of 2.2, a specificity of 56.2%, and an LR (−) value of 0.07. With respect to BF% among the women in the study population, the cut-off point value of 38.9% provided a sensitivity of 97.4%, an LR (+) value of 2.2, a specificity of 55.9%, and an LR (−) value of 0.04. The ROC curve for FMI was also obtained, using a cut-off value of 11.8 kg/m^2^, a sensitivity of 97.6%, an LR (+) of 2.2, a specificity of 56.9%, and an LR (−) of 0.04.

## 4. Discussion

In our study, we found that the MetS prevalence was higher in men than in women (11.2% vs. 5.3%; *p* < 0.001), which is an intermediate value compared to those reported in local and international studies, ranging from 2 to 13% [31,32,33], that BF% and FMI were positively correlated to MetS components (*p* < 0.05), and that the ROC analysis showed that both the BF% and FMI had a moderate discriminatory power in the identification of MetS among Colombian university students.

The higher prevalence of MetS, at 11.2%, seen in male subjects in our results does not coincide with Ruano-Nieto et al. [34] who found that the estimated prevalence of MetS was 8.4% for women and 6.1% for men in a population of Ecuadorian university students. The overall prevalence of MetS in our study, at 7.7%, was also greater than that in other Latin American countries such as Chile at 4.9% and Argentina at 4.1% [35,36]. Clearly, the prevalence of MetS could differ between studies depending on the MetS cluster used, the design method, and the target population. In this study, we used the IDF and AHA/NHLBI [2] joint statement, as it was an international attempt to harmonize the definition of MetS; central obesity is not an obligatory component of this definition and it is ethnic-specific.

Of those components, abdominal obesity, the most prevalent manifestation of MetS, is a marker of dysfunctional adipose tissue, and is of central importance in clinical diagnosis [37,38,39,40,41]. Our results show correlation between BF%, FMI, and all of the cardiometabolic biomarkers analyzed, including mean arterial pressure, glucose, HDL-C, LDL-C, triglycerides, and total cholesterol (<0.05 for all). These findings agree with Knowles et al. [19], who studied a population of young Peruvian adults and found significant correlations between the fat parameters and the above-mentioned cardiometabolic biomarkers. Blood lipid disorders and adipose tissue are the key etiologic defects that define MetS; we found all fat indices used in this study were associated with BF%. In contrast, other researchers such as Schuster et al. [40], who studied a sample of 444 young adults in Brazil, only found correlations between the BF% and glucose, HDL-C, and triglycerides. Our result may be different due to degree and the prevalence varying on the basis of ethnicity, genetic susceptibility, lifestyle, and geographic location. In addition, our results showed lower values of weight, height, and WC in women compared to men. This aligns with Liu et al. [17], who studied a population of 1698 adults in China. In all likelihood, these differences in body composition were due to sexual dimorphism [31] and diet [34]. Sex-specific hormones are another possible explanation. For example, the triglyceride levels and blood pressure were also lower among women in comparison to men. In this context, much of the risk of MetS associated with sex can be explained by the change in steroid hormone levels and the metabolism of carbohydrates and lipids [39]. An increase in total fat and the distribution of central fat, resulting from alterations in biomarker-disease, such as fasting glucose, triglycerides, and ferritin, which are mediators of MetS, has been reported in female Hispanic/Latino adults [40]. This coincides with previous research, in which this finding was correlated with lower cardiovascular morbidity and dysfunctional adipose tissue in women [39,41]. Furthermore, the observation of ongoing changes in body composition by sex and its relationship with obesity has demonstrated that obesity affects individuals no matter their muscle mass, fat mass, height, and weight [41].

The AUC values for the MetS ROC analyses, at 0.835 for men and 0.838 for women, indicate that BF% has moderate diagnostic capabilities to identify subjects with MetS (≥3 risk factors). Interestingly, when considering MetS in our study, FMI showed the greatest AUC to predict MetS risk in women compared to men. Results show that metabolic disorders are present in young adult independent of age, smoking, physical activity, or fitness levels [42]. There is a growing interest in suggesting cut-off points for body fat levels for the early detection of CVD risk. Future longitudinal studies should be carried out to understand the role of different levels of adiposity on CVD risk stratification within a college population.

In contrast, another study carried out by Mohammadreza et al. [43] among Iranian adults found that BF% was not as effective a parameter for predicting MetS as other anthropometric indexes such as BMI, waist-to-height ratio, and waist-to-hip ratio. In this same line, Mousa et al. [44] studied a population of young adults and found that the predictive power of BF% and BMI for screening MetS was limited. This discrepancy in results could be explained by the heterogeneity of the sample populations. Consequently, the applicability and usefulness of BF% and FMI in the prediction of MetS require further studies of different populations and ethnic groups. These results are partially consistent with previous studies that showed BF% was positively correlated with single metabolic risk factors, such as BMI and triglycerides, and negatively correlated with HDL cholesterol in men, but not in women [45,46].

To our knowledge, few studies have explored the use of FMI as a proxy of obesity. In a study of 538 Mexican American college students (373 women and 165 men), the validity of FMI and BMI against BF% with BIA as the reference method was evaluated [47]. Correlations between FMI and BF% resulted in correlation coefficients of 0.976 in men (*p* < 0.001) and 0.992 in women (*p* < 0.001). However, similar to our findings, the correlations between FMI and PBF were higher (*r* = 0.960, *p* < 0.01). In a study of 2986 healthy white men and 2649 white women, age 15 to 98 years in Switzerland, the mean FMI in the overall age group was 4.9 (1.8) kg/m^2^ in men and 6.6 (2.4) kg/m^2^ in women [48], compared to our results, of 6.6 (3.1) kg/m^2^ in men and 11.2 (3.4) kg/m^2^ in women without MetS. This difference can be explained by the fact that FMI cutoffs in the Swiss group were derived from BMI and not from MetS cutoffs. On the other hand, in the Peltz et al. study [47], the median FMI in the overall age group was 7.3 (interquartile range 4.5) kg/m^2^ in men and 9.0 (interquartile range 5.9) kg/m^2^ in women. Our FMI means fell within the lower range of the Mexican American normal FMI ranges in both men and women. This was expected, as South Americans have a lower percentage of body fat across all age groups when compared to non-Hispanic whites, Mexican Americans, and non-Hispanic blacks [49]. In a subsequent study from the same group (5635 apparently healthy adults from a mixed non-randomly selected Caucasian population) in Switzerland, obesity was defined as an FMI greater than 8.2 kg/m^2^ in men and 11.8 kg/m^2^ in women [50], which are similar than our FMI cutoffs of ≥6.9 kg/m^2^ in men and ≥11.8 kg/m^2^ in women in our study. These findings, although limited, show an interesting agreement with our results, which collectively contribute to the concept of developing one set of recommended ranges that are not affected by height.

Our results have public health and clinical implications as college populations have been reported as a period in life when several behavioral and metabolic changes occur. These changes, in addition to the adoption of a Western lifestyle and diet, have led to a rise in the prevalence of overweight and obese Colombians, particularly among university students [32,51]. Further studies are needed to identify clinical characteristics in young adults that could be used in screening tests to predict MetS risk in adulthood.

The principal limitation of this study was the use of cross-sectional data. Furthermore, it is also true that the hydration status of the participants could have modified the results of the BF% measured with BIA. Most BIA prediction equations assume that the fat mass and fat-free mass consist of 73% water [52]. These major limitations of the BIA method remain unresolved. Secondly, the assessment of other biomarkers, such as insulin, adiponectin, and c-reactive protein levels, which could have shown additional information about prognostic assessment, was not performed. These and other questions deserve further investigation by future well-designed longitudinal studies.

The main strength of our study is the fact that our study compared the predictive power of both BF% and FMI while providing cutoff values for the prediction of MetS in university students from Colombia. Considering the wide use of BIA methods and their extension to assess body composition, our study has important implications for evaluating CVD and metabolic risks in high-risk subjects, such as those with MetS.

## 5. Conclusions

In conclusion, BF% and FMI had a moderate discriminatory power in the identification of MetS in Colombian university students. Apart from the differences between our cut-off points and those reported in other research in geographically different populations, this study reports the first cutoff points for identification of MetS by BF% and FMI in Colombian young adults. We demonstrated that a BF% ≥38.9% in women and ≥25.5% in men, and an FMI ≥11.8 kg/m^2^ in women and ≥6.9 kg/m^2^ in men, are thresholds that could be used to predict Colombian young adults at high risk of MetS.

## Figures and Tables

**Table 1:** Characteristics among a sample of college students from Colombia (mean (SD) or frequency (%)).

Characteristic	Men (*n* = 617)	Women (*n* = 1070)	*p*-Value
Anthropometric			
Age (years)	20.6 (2.2)	20.6 (2.0)	0.843
Weight (kg)	58.9 (10.0)	69.9 (12.4)	<0.001
Height (cm)	159.8 (6.1)	172.5 (6.7)	<0.001
WC (cm)	78.4 (9.5)	71.5 (7.9)	<0.001
BMI (kg/m^2^)	23.2 (3.7)	23.2 (3.7)	0.356
Body fat (%)	15.7 (6.7)	27.0 (7.2)	0.028
FMI	3.9 (2.3)	6.5 (2.7)	<0.001
Body mass index status *n* (%) *			
Underweight	34 (5.5)	71 (6.7)	<0.001
Normal weight	425 (68.8)	725 (57.8)	
Overweight	128 (20.8)	220 (20.5)	
Obese	31 (5.0)	54 (5.0)	
Blood pressure			
Systolic blood pressure (mmHg)	120.83 (13.0)	111.28 (11.1)	<0.001
Diastolic blood pressure (mmHg)	74.83 (11.4)	71.78 (9.3)	<0.001
Mean arterial pressure (mmHg)	97.83 (10.8)	91.53 (8.9)	<0.001
Metabolic biomarkers			
Total cholesterol (mg/dL)	132.26 (30.8)	146.27 (33.3)	0.212
Triglycerides (mg/dL)	93.01 (48.7)	88.10 (43.7)	0.011
LDL-C (mg/dL)	38.92 (10.4)	43.98 (12.8)	<0.001
HDL-C (mg/dL)	81.89 (26.5)	87.85 (26.2)	0.589
Glucose (mg/dL)	84.36 (12.2)	85.99 (11.6)	0.002
Metabolic Syndrome *n* (%) *			
Yes	73 (11.2)	59 (5.3)	0.001
Life-style *n* (%) *			
Tobacco (≥10 cigarettes per week)	183 (29.7)	213 (19.9)	0.289
Alcohol (≥1 times per week)	294 (47.6)	381 (35.6)	0.358
PA (three or more times a week for >30 min)	213 (34.5)	228 (21.3)	0.011

Continuous variables are reported as mean values (standard deviations) and categorical variables are reported as numbers and percentages in brackets. Significant between-sex differences (*t*-tests or * chi-square test χ^2^). WC: waist circumference; BMI: body mass index; FMI: fat mass index; LDL-C: low-density lipoprotein cholesterol; HDL-C: high-density lipoprotein cholesterol; PA: physical activity.

**Table 2:** The descriptive characteristics of participants with and without MetS in both sexes.

Variable	Men (*n* = 617)			Women (*n* = 1070)		
	MetS (*n* = 73)	Non-MetS (*n* = 544)	*p* Value	MetS (*n* = 59)	Non-MetS (*n* = 1011)	*p* Value
Anthropometric						
Age (years)	21.7 (3.4)	20.4 (3.1)	0.229	22.3 (3.8)	20.5 (2.8)	<0.001
Weight (kg)	80.8 (15.8)	67.3 (10.7)	<0.001	76.4 (13.8)	57.4 (8.8)	<0.001
Height (cm)	172.7 (7.5)	172.1 (6.6)	0.129	161.1 (5.6)	158.9 (5.8)	0.902
WC (cm)	89.5 (11.6)	76.7 (8.0)	<0.001	84.9 (8.8)	70.5 (7.1)	0.145
BMI (kg/m^2^)	27.0 (4.7)	22.6 (3.1)	<0.001	29.3 (4.7)	22.7 (3.3)	<0.001
Body fat (%)	23.5 (7.5)	14.5 (5.7)	0.005	37.3 (6.0)	26.2 (6.8)	<0.001
FMI (kg/m^2^)	6.6 (3.1)	3.4 (1.8)	<0.001	11.2 (3.4)	6.1 (2.4)	<0.001
Body mass index status *n* (%) *						
Underweight	4 (0.6)	30 (5.0)	<0.001	0.0 (0.0)	71 (6.6)	<0.001
Normal weight	16 (2.6)	408 (66.1)		8 (0.8)	717 (67.0)	
Overweight	37 (6.0)	91 (14.8)		23 (2.1)	197 (18.4)	
Obese	16 (2.6)	15 (2.4)		28 (2.6)	26 (2.4)	
Blood pressure						
Systolic blood pressure (mmHg)	131.01 (11.96)	119.36 (12.44)	0.461	123.50 (11.03)	110.33 (10.64)	0.809
Diastolic blood pressure (mmHg)	83.60 (10.75)	73.48 (10.07)	0.160	81.61 (13.82)	71.10 (8.60)	0.237
Mean blood pressure (mmHg)	107.30 (10.20)	96.42 (10.07)	0.176	102.55 (9.16)	90.71 (8.36)	0.518
Metabolic biomarkers						
Total cholesterol (mg/dL)	146.01 (39.6)	130.27 (29.1)	<0.001	153.39 (33.7)	145.74 (33.3)	0.955
Triglyceride (mg/dL)	163.03 (75.9)	83.04 (33.7)	<0.001	139.83 (66.6)	84.90 (40.2)	<0.001
HDL-C (mg/dL)	31.27 (5.9)	40.06 (10.5)	<0.001	36.51 (9.0)	44.52 (12.9)	0.002
LDL-C (mg/dL)	86.01 (30.8)	81.31 (26.0)	0.077	89.61 (28.4)	87.57 (26.1)	0.571
Glucose (mg/dL)	92.44 (13.8)	83.14 (11.6)	0.063	92.58 (14.4)	85.49 (11.3)	0.142
Life-style *n* (%) *						
Tobacco (≥10 cigarettes per week)	19 (26.0)	155 (28.5)	0.769	5 (8.5)	198 (19.6)	0.088
Alcohol (≥1 times per week)	36 (49.3)	294 (54.0)	0.563	22 (37.3)	394 (38.7)	0.378
PA (three or more times a week for >30 min)	13 (17.81)	188 (34.5)	0.005	4 (6.8)	213 (21.0)	0.018

Continuous variables are reported as mean values (standard deviations) and categorical variables are reported as numbers (percentages). Significant between-sex differences (*t*-tests or * chi-square test χ^2^). WC: waist circumference; BMI: body mass index; FMI: fat mass index; LDL-C: low-density lipoprotein cholesterol; HDL-C: high-density lipoprotein cholesterol; PA: physical activity. The mean blood pressure was calculated using the following formula: (systolic blood pressure + (2 × diastolic blood pressure))/3.

**Table 3:** Results of the partial correlation analysis between body fat percentage (BF%), fat mass index (FMI), and MetS components.

Variable	Glucose (mg/dL)	HDL-C (mg/dL)	Triglycerides (mg/dL)	Total Cholesterol (mg/dL)	MAP (mmHg)	WC (cm)	FMI (kg/m^2^)
Body fat (%)	0.188 *	−0.239 **	0.230 **	0.279 **	0.276 **	0.827 **	0.960 **
FMI (kg/m^2^)	0.113 **	−0.256 **	0.230 **	0.162 *	0.272 **	0.860 **	1
WC (cm)	0.106 *	−0.219	0.248	0.858 **	0.271 **	1	
Mean blood pressure (mmHg)	0.004	−0.022 **	0.179 **	0.259 **	1		
Total cholesterol (mg/dL)	−0.008	−0.341 **	0.241 **	1			
Triglycerides (mg/dL)	0.125 **	−0.170 **	1				
HDL-C (mg/dL)	−0.158 **	1					
Glucose (mg/dL)	1						

Analysis adjusted by co-variables: age, sex, tobacco, alcohol, and physical activity. * *p* < 0.05; ** *p* < 0.01. WC: waist circumference; FMI: fat mass index; HDL-C: high-density lipoprotein cholesterol; MAP: mean arterial pressure. The mean blood pressure was calculated using the following formula: (systolic blood pressure + (2 × diastolic blood pressure))/3.

**Table 4:** Parameters of the ROC curves analysis for the diagnostic performance of body fat percentage (BF%) and fat mass index (kg/m^2^) in identifying high risk of MetS according to the IDF criteria in men and women.

Parameter			BF%	FMI
High risk of MetS	Men	AUC	0.835	0.838
		95% CI	0.779–0.891	0.779–0.892
		*p* value	<0.001	<0.001
		Cut-off	25.5	6.9
		Sensitivity (%)	96.1	95.8
		Specificity (%)	57.5	56.2
		LR (+)	2.3	2.2
		LR (−)	0.06	0.07
	Women	AUC	0.887	0.889
		95% CI	0.842–0.932	0.844–0.933
		*p* value	<0.001	<0.001
		Cut-off	38.9	11.8
		Sensitivity (%)	97.4	97.6
		Specificity (%)	55.9	56.9
		LR (+)	2.2	2.2
		LR (−)	0.04	0.04

AUC: area under the curve; CI: confidence interval; LR (+): positive likelihood ratio; LR (−): negative likelihood ratio.

## Abbreviations

The following abbreviations are used in this manuscript:

BF	body fat
BIA	bioelectrical impedance analysis
BMI	body mass index
CI	confidence interval
CT	computed tomography
CVD	cardiovascular disease
DXA	dual-energy x-ray absorptiometry
FUPRECOL	association between muscular strength and metabolic risk factors in colombia
FMI	fat mass index
HDL-C	high-density lipoprotein cholesterol
IDF	international diabetes federation
LDL-C	low-density lipoprotein cholesterol
MetS	metabolic syndrome
MRI	magnetic resonance imaging
PA	physical activity
SD	standard deviation
WC	waist circumference
WHO	World Health Organization
